# An entertainment-education approach to prevent COVID-19 spread: study protocol for a multi-site randomized controlled trial

**DOI:** 10.1186/s13063-020-04942-7

**Published:** 2020-12-15

**Authors:** Alain Vandormael, Maya Adam, Merlin Greuel, Till Bärnighausen

**Affiliations:** 1 Heidelberg Institute of Global Health, University of Heidelberg, Heidelberg, Germany; 2 Department of Pediatrics, Stanford University School of Medicine, Stanford, CA USA; 3 Department of Global Health and Population, Harvard T. H Chan School of Public Health, Boston, USA; 4 Africa Health Research Institute (AHRI), Somkhele, KwaZulu-Natal South Africa

**Keywords:** COVID-19, Randomized controlled trial, Protocol, Entertainment-education, Behavioral intent, Knowledge, List experiment

## Abstract

**Background:**

Entertainment-education (E-E) media can improve behavioral intent toward health-related practices. In the era of COVID-19, millions of people can be reached by E-E media without requiring any physical contact. We have designed a short, wordless, animated video about preventive COVID-19 behaviors that can be rapidly distributed through social media channels to a global audience. The E-E video’s effectiveness, however, remains unclear.

**Methods/design:**

This is a multi-site, parallel group, randomized controlled trial comparing the effectiveness of an E-E video on COVID-19 against (i) an attention placebo control (APC) video and (ii) no video. For our primary outcomes, we will measure knowledge about preventive COVID-19 behaviors. We will also use a list randomization approach to measure behavioral intent toward preventative COVID-19 behaviors. In each trial arm, participants will be randomized to a control list or a control list plus an item about social distancing, washing hands, cleaning household surfaces, sharing of eating utensils, and the stockpiling of essential goods. Using an online platform, we will recruit 17,010 participants (aged 18–59 years) from the USA, the UK, Germany, Spain, France, and Mexico.

**Trial registration:**

German Clinical Trials Register #DRKS00021582. Registered on May 12, 2020.

**Discussion:**

This trial will utilize several randomization procedures, list experimentation methods, and state-of-the-art online technology to demonstrate the effectiveness of an E-E video to improve knowledge of, and behavioral intent toward, the prevention of COVID-19. Our results will inform future E-E video campaigns for COVID-19 and similar public health intervention needs.

## Administrative information

**Table:** 

**Title**	An entertainment-education approach to prevent COVID-19 spread: study protocol for a multi-site randomized controlled trial
**Trial registration**	The study and its outcomes were registered at the German Clinical Trials Register (www.drks.de) on May 12th, 2020: #DRKS00021582.
**Protocol version**	1.1, 4 June 2020.
**Funding**	This study is funded by an Alexander von Humboldt University Professor Prize awarded to Dr. Till Bärnighausen.
**Author details**	1 Heidelberg Institute of Global Health, University of Heidelberg, Heidelberg, Germany 2 Department of Pediatrics, Stanford University School of Medicine, Stanford, CA, USA 3 Department of Global Health and Population, Harvard T. H Chan School of Public Health, Boston, USA. 4 Africa Health Research Institute (AHRI), Somkhele, KwaZulu-Natal, South Africa
**Name and contact information for the trial sponsor**	Not applicable
**Role of sponsor**	Not applicable

## Background and rationale

A large amount of information about novel coronavirus (COVID-19) has been disseminated by the traditional mass media since the outbreak of the pandemic [1–3]. However, it is not clear if this dissemination has improved knowledge of, or intent toward, preventive COVID-19 behaviors. By preventive behaviors, we mean the public’s adoption of practices, such as social distancing, reduced physical contact, and hand/surface sanitization (among others), to reduce the spread of COVID-19.

Arguably, one possible limitation of mainstream mass media is that it has been perceived as politicized and culturally localized, thus limiting the persuasiveness of messages about COVID-19 prevention [4, 5]. It is also likely that traditional mass media channels (e.g., local or national television networks) have missed key segments of the population, such as young people (25–40 years) who are disproportionately more likely to transmit the virus to older people (≥ 50 years) [6]. An effective public health response could therefore benefit from entertainment-education (E-E) approaches that increase COVID-19 prevention outcomes [7].

To improve knowledge and behavioral intent toward COVID-19 prevention, we have designed an animated, wordless, E-E video that can be rapidly distributed to a diverse and global audience through social media channels. With a short duration (approximately 2.30 min), the E-E video contains no speech and minimizes cultural signifiers to increase universality and appeal. The E-E video was released on Stanford Medicine’s YouTube channel on March 21, 2020, and went viral within 24 h. After 10 days, it had reached 332,000 views on YouTube, 220,000 views on Instagram, 294,000 views on Facebook, and 402,000 views on Twitter, with a cumulative count of 1.2 million views [8]. The E-E video could play a useful role in disseminating evidence-based health recommendations related to COVID-19; however, its effectiveness remains unclear.

### Objectives

The study aims to achieve the following objectives. To establish:

1. The E-E video’s effectiveness in improving knowledge of preventive COVID-19 behaviors
2. The E-E video’s effectiveness in increasing behavioral intent toward COVID-19 prevention
3. People’s voluntary interest to watch the E-E video

### Trial design

**Fig. 1:**
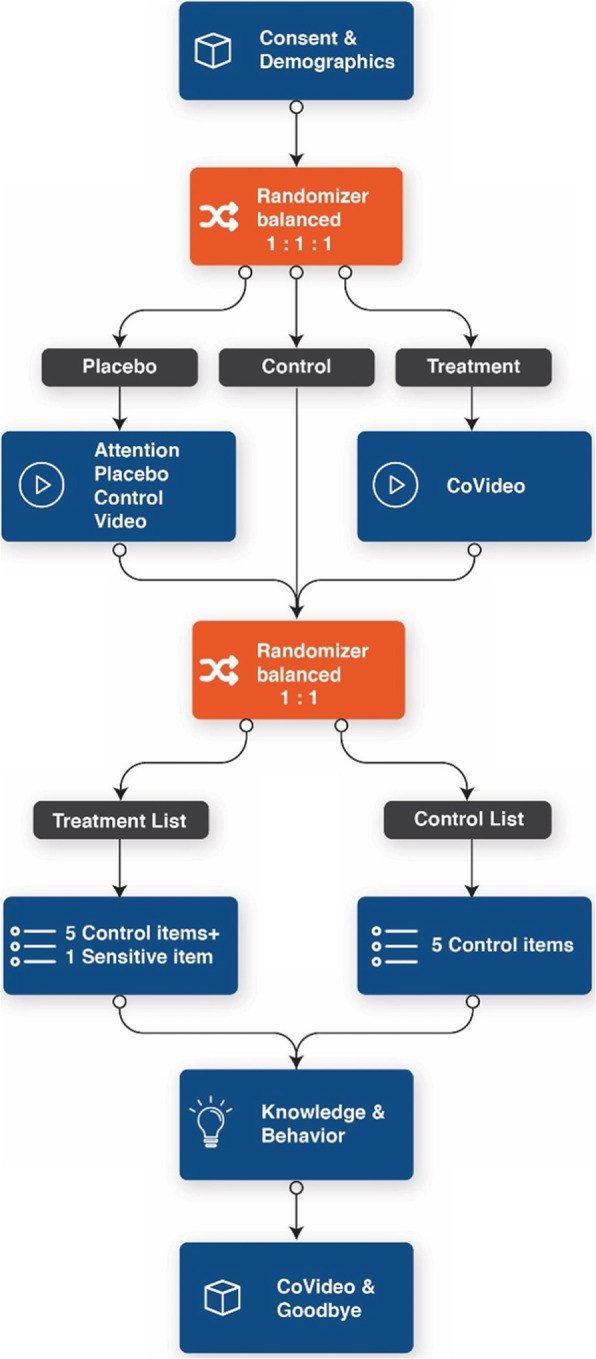
The trial design, which consists of three arms. Participants will be randomly assigned (at a 1:1:1 ratio) to the E-E video (CoVideo, arm 1), an APC video (arm 2), or no video (arm 3). Each arm has a list experiment, with participants split (at a 1:1 ratio) into a control or treatment group

The present study is a multi-site, parallel group, randomized controlled trial (RCT) comparing the effectiveness of the E-E video against an APC video or no video (Fig. 1) [9]. Randomization is at a 1:1:1 ratio for the three arms of the trial. Nested within each arm of the trial is a list experiment. Participants will be randomized at a 1:1 ratio to a control or treatment list. After completing the list experiment, participants in arms 2 and 3 will receive the E-E video to ensure post-trial access to treatment [10].

## Methods: participants, interventions, and outcomes

### Study setting

**Fig. 2:**
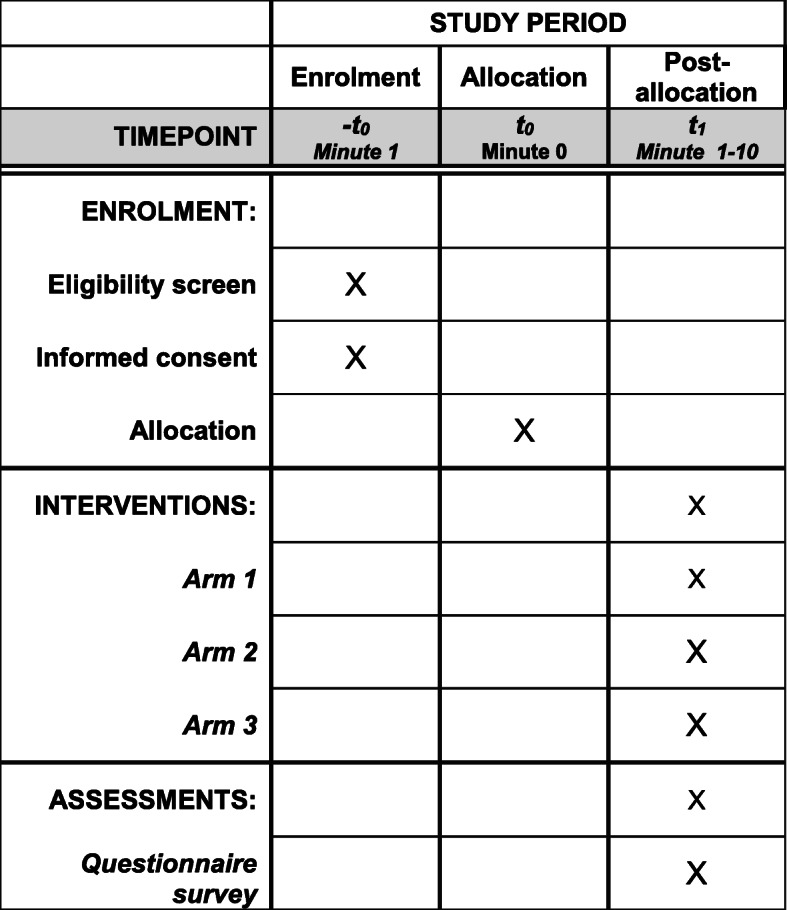
Schedule of enrolment, interventions, and assessments for the study

This will be an online study setting. We will use the online recruitment platform Prolific Academic (ProA: https://www.prolific.co) to enroll participants from the USA, the UK, Germany, Spain, Mexico, and France. We will host and deploy our study on an online platform called Gorilla™ (www.gorilla.sc) (Fig. 2).

### Eligibility criteria

Registered participants on the ProA platform must be between the ages of 18 and 59 years (male, female, or other), have current residence in the target countries, and speak English, German, or Spanish.

### Ethical approval

Ethical approval was obtained from the Stanford University IRB on April 12, 2020, protocol #55820.

### Who will take informed consent?

Participants will undergo a process of informed consent on the ProA platform. The consent form explains the purpose of the study, the risks and benefits of the research, and how to contact the study investigators (or the Stanford University ethics review board) about problems experienced during the course of the study.

### Criteria for discontinuing or modifying allocated interventions

We will not discontinue or modify the allocated interventions during the course of the study. Participants may choose to discontinue their participation at any time.

## Interventions

### Intervention description

The intervention is an E-E video about COVID-19 prevention, which we abbreviate to CoVideo. Developed by our co-author (MA) for Stanford Medicine, the CoVideo is animated with sound effects and has no words, speech, or text. The CoVideo shows how the novel coronavirus is spread (airborne, physical contact) and recommends best practices to prevent onward transmission (staying at home, not congregating in public spaces, and sanitizing hands/surfaces). The CoVideo also covers the mass media coverage of the outbreak and the public’s response to this media coverage, which includes a subplot on the stockpiling of essential goods, and the impact thereof on healthcare services and resources (e.g., doctors being unable to access protective equipment). The CoVideo can be viewed at https://www.youtube.com/watch?v=rAj38E7vrS8.

### Explanation for the choice of comparators

The comparators are an APC video (arm 2) or no video (arm 3). The APC video is similar in style to the CoVideo; it is also animated with a duration of 2.30 min and has sound effects but no words, speech, or text. The video message is about how small choices become actions, which become habits, which become a way of life (https://www.youtube.com/watch?v=_HEnohs6yYw). The APC will enable us to quantify the attention effect of the CoVideo. APC conditions should mimic the “inactive” components of an intervention—the effect of watching the video—while not containing any of the “active” intervention components—the content delivered by the video [11]. To measure its total effect, we also compare the CoVideo with a control (no video). We do not assume the CoVideo is better than nothing; it is possible that the CoVideo could motivate reactance to our message about COVID-19 prevention [12–14]. For the list experiments, we will use the control list as the comparator.

### Outcomes

#### Primary outcome measures

There are two primary outcome measures. First, we will use 18 True/False questions to assess if the CoVideo improves knowledge about preventive COVID-19 behaviors. These questions are shown in Table 1 and this data will be used to assess objective 1.

**Table 1:** The COVID-19 knowledge items, which require True/False responses

The current coronavirus can be spread by an infected person even if they look healthy	Cleaning surfaces with soap and water is an effective way to kill the current coronavirus
The current coronavirus cannot be spread from person to person	An effective way to prevent COVID-19 spread is to wear a face mask even if you do not have COVID-19 symptoms
The current coronavirus cannot survive on surfaces for more than a few minutes	An effective way to prevent COVID-19 spread is to wash your hands frequently with soap and water
Some people with COVID-19 infection may experience a cough	An effective way to prevent COVID-19 spread is to regularly rinse your nose with salt water
Some people with COVID-19 infection do not experience a fever	An effective way to prevent COVID-19 spread is to avoid touching your face
The current coronavirus spreads from person to person through small droplets from the mouth	An effective way to prevent COVID-19 spread is to avoid shaking hands with other people
The current coronavirus spreads from person to person through small droplets from the nose	An effective way to prevent COVID-19 spread is to avoid places that are crowded with people (like bars, restaurants, or performances)
You can catch COVID-19 by touching a contaminated surface and then touching your face	An effective way to prevent COVID-19 spread is to eat garlic with each meal
Antibiotics can be used to treat COVID-19 infection	An effective way to prevent COVID-19 spread is to avoid sharing eating utensils with others

Second, we will assess if the CoVideo improves behavioral intent toward preventive COVID-19 behaviors. Since many participants will be primed to give socially desirable answers to the behavioral intent questions, we will use a list randomization approach [15–17]. There will be five list experiments, which are shown in Table 2. For each experiment, the control group will receive a list of five items. Participants will be asked to state *how many* of the items they agree with, without stating which ones they agree with. For the first list experiment, imagine that the control group selects an average of 2 out of the 5 items. The treatment group will get the same list but with one additional “sensitive” item about behavioral intent. Using the example, the participants in the treatment group select an average of 2.2 out of the 6 items. Holding all else equal, we conclude that the prevalence of participants that would go out with friends (item 6 of list 1 in Table 2), in defiance of lockdown regulations, is 20%. We designed the list experiments in line with best practices [17], and we will use this data to assess objective 2.

**Table 2:** In each trial arm, both groups will receive five lists. For each list, the control group will get the first five items only; the treatment group will receive the five items and the sixth sensitive item, indicated by an asterisk (*). Each list experiment will be preceded by the question: “How many of the five/six statements do you agree with? We don’t want to know which ones, just answer how many. This week I will spend time watching TV, etc.”

**List 1: Social distancing**	**List 2: Wash hands**
1. Spend time watching TV	1. Clip my toenails
2. Do the vacuuming	2. Spend time watching movies
3. Pick a fight with my partner	3. Clean the toilet
4. Eat a low sugar diet	4. Smoke marijuana
5. Rinse my nose with salt water daily	5. Eat fruit daily
6. Go out with my friends*	6. Wash my hands frequently*
**List 3: Clean surfaces**	**List 4: Share utensils**
1. Watch a new TV series	1. Spend time on the Internet
2. Spend time gardening by myself	2. Do daily indoor exercises
3. Try to go vegetarian	3. Take an online course
4. Have alcoholic drinks on at least three evenings	4. Play a prank on my partner
5. Catch up on last week’s work	5. Smoke cigarettes
6. Clean kitchen counters after use*	6. Clean my dishes after use*
**List 5: Stockpiling**	
1. Spend time chatting with my friends online	
2. Try new cooking recipes	
3. Watch a pornographic movie	
4. Clean all floor surfaces	
5. Visit the World Health Organization (WHO) website	
6. Stock up on household supplies for a month*	

#### Secondary outcome measure

We will assess participant’s behavioral intent to seek health information from online, animated videos. Because of social desirability bias, we will again use a list experiment with the same procedures described in Table 2. The list experiment will read: This week, I will seek health information from: Television news channels; Social media celebrities; Religious leaders on the Internet; Public health agency websites; Conversations with family and friends; *Animated videos made by health experts. (The sensitive item is indicated by an asterisk (*).)

We aim to measure participant engagement with the CoVideo. At the end of the survey, we will offer the APC and no-video arms the choice to watch the CoVideo or end the survey. The Gorilla platform will record this response. If the “Watch Video” button is clicked, Gorilla will record the time (in milliseconds) from the video start until the participant clicks the “Finish” button (or until the video ends at 2.30 min, whichever comes first). This data will be used to assess objective 3.

### Sample size

We calculated the sample size needed for pairwise comparisons between three groups using a one-way analysis of variance (ANOVA). The formula to calculate the sample size is [18]:

$$ {n}_A=\left({\sigma}_A^2+{\sigma}_B^2/\kappa \right){\left(\frac{Z_{1-\frac{\alpha }{\tau }}+{Z}_{1-\beta }}{\mu_A-{\mu}_B}\right)}^2 $$

where *κ* = 1, which is the matching ratio; *μ*_*A*_ and *μ*_*B*_ are the group A and B means; *σ*_*A*_ and *σ*_*B*_ are the group A and B standard deviations; *α* = 0.05 is the type-I error; *β* = 0.20 is the type-II error; *Z* is the quantile function; and *τ* = 2 is the number of comparisons to be made. To detect a small difference of 0.1 between the knowledge scores of two trial arms, we assumed a mean of *μ*_*A*_ = 14.0 and *μ*_*B*_ = 14.1 and *σ*_*A*_ = 1.5 and *σ*_*B*_ = 1.5. We select *μ* on the expectation that participants will get 14 out of 18 knowledge items correct. This gives a sample size of *n*_*A*_ = *n*_*B*_ = *n*_*C*_ = 3,532, so *N* = 10,596. If we change our assumption about *σ* and increase it to *σ* = 1.8, then the required sample size becomes *n*_*A*_ = *n*_*B*_ = *n*_*C*_ = 5,087 and *N* = 15,261. For the list experiment, we selected a sample size to detect a small difference of 0.05 between the control list group and the treatment list group. Because we framed the list questions to avoid floor and ceiling effects, we expect, on average, that the control group will agree with 2 out of the 5 items and the treatment group 2.05 of the 6 items. We selected *σ*_*A*_ = 0.7 and *σ*_*B*_ = 0.8, allowing the second *σ* to be larger because of the additional sensitive item. This calculation gives a sample size of *n*_*A*_ = *n*_*B*_ = *n*_*C*_ = 3,548 and *N*= 10,644. If we change our assumption about *σ* and increase it to *σ*_*A*_ = 0.9 and *σ*_*B*_ = 1.0, then we have *n*_*A*_ = *n*_*B*_ = *n*_*C*_ = 4,553 and *N* = 13,659. For this study, we will recruit 17,010 participants, more than the 15,261 calculated sample size, to ensure our study is sufficiently powered.

### Recruitment

To be recruited, a person must open an account on ProA and provide his or her personal information. Participants must agree to ProA’s data privacy terms and conditions. ProA will assign each participant a unique, anonymized ID. The study investigators will also open an account on ProA. We will instruct the ProA platform on how many participants need to be recruited. ProA will filter out all participants who do not meet the eligibility criteria. Participant recruitment will happen on a “first come, first served” basis until the recruitment number (sample size) is reached. We will compensate the participants an equivalent of £1 for the expected 10-min completion time.

### Assignment of interventions: allocation

The Gorilla platform is designed to host and implement online experimental studies (implementation). Gorilla will randomly allocate participants to the intervention (CoVideo), placebo (APC), or control (no video) arm (sequence generation). Gorilla will use a web-based randomization algorithm, which is unknown to us (concealment mechanism).

### Assignment of interventions: blinding

#### Who will be blinded

The study investigators and those involved in the data analyses and statistics will be blinded to the group allocation.

### Data collection and management

#### Plans for assessment and collection of outcomes

Data will be collected on the Gorilla platform. Participants will submit data by clicking on the response buttons. We expect to collect the data over a 4-week period.

#### Plans to promote participant retention and complete follow-up

Participants will automatically exit the study if they take longer than 45 min to complete the survey. The time-out is to ensure that participants do not clog up the system with incomplete surveys. Since the participants are anonymous to us, there is no way to initiate follow-up in the time limit.

#### Data management

Gorilla will store the trial data on its cloud platform, hosted on Microsoft Azure in the Republic of Ireland. The Gorilla database is encrypted using industry-standard cryptography. The study investigators own the research data that has been collected using Gorilla and have complete control over it. The study investigators can generate and access the completely anonymized data from the Gorilla platform. The data will be downloaded and safely stored for statistical analysis on a computing system maintained by the University of Heidelberg in Germany.

#### Confidentiality

The participants, who are completely anonymous to us, will have no identifying information associated with their unique IDs. We will inform participants that if they email the study investigators then their names could be revealed to us. The study investigators will keep this information confidential.

### Statistical methods

#### Descriptive measures

We will use descriptive statistics to obtain the mean and standard deviation of age, the proportion of participants by gender, and frequency distributions for country of residence and education status.

#### Primary outcomes

For each participant, we will sum the knowledge items that are correctly answered (min. = 0, max. = 18). We will then obtain the mean knowledge score for each trial arm. Let ${\overline{K}}_k$ denote the mean knowledge score for the *k*th trial arm, where *k* ∈ {*v*, *a*, *c*} such that *v* represents the CoVideo, *a* represents the APC, and *c* represents the control. We will use an ordinary least squares (OLS) regression model to estimate ${\overline{K}}_k$ as well as obtain the difference estimators. We define the difference between the CoVideo (${\overline{K}}_v\Big)$ and the control (${\overline{K}}_c\Big)$ as the total effect, the difference between the CoVideo (${\overline{K}}_v\Big)$ and the APC (${\overline{K}}_a\Big)$ as the content effect, and the difference between the APC video (${\overline{K}}_a\Big)$ and the control (${\overline{K}}_c\Big)$ as the attention effect.

For each list experiment, we will calculate the mean score for the control list, denoted by $\overline{C_i}$, and the mean score for the treatment list, denoted by $\overline{T_i}$, for the *i*th list experiment (*i* = 1, …, 5). We will then estimate the difference between the treatment and control list for each trial arm, denoted by ${D}_{ik}={\overline{T}}_{ik}-{\overline{C}}_{ik}$, for the *i*th treatment list and *k*th trial arm, where *k* ∈ {*v*, *a*, *c*}. Let $D{D}_i^T$ denote the total effect, which is estimated by *D*_*iv*_ − *D*_*ic*_; let $D{D}_i^C$ denote the content effect, which is estimated by *D*_*iv*_ − *D*_*ia*_; and let $D{D}_i^A$ denote the attention effect, which is estimated by *D*_*ia*_ − *D*_*ic*_. These analyses are analogous to difference-in-difference analyses, which can be implemented by specifying the appropriate main and interaction terms in an OLS regression.

#### Secondary outcomes

Using the same statistical procedure described above, we will assess behavioral intent to seek health information with E-E media. To establish participant engagement with the CoVideo, we will use a logistic regression model to estimate the proportion of participants in the APC and control arms that chose to watch the CoVideo, adjusting for the knowledge scores and demographic factors. We will use either an OLS model or Cox proportional hazards model to model the time to when a person clicks “Finish Video” (right censoring) or when the CoVideo ends naturally (left censoring). We will use R statistical software to undertake the analysis.

#### Interim analyses

No interim analyses are planned.

#### Methods for additional analyses (e.g., subgroup analyses)

We will conduct both country-specific and cross-country pooled analyses, provided sufficient sample sizes can be recruited from each country.

#### Methods in analysis to handle protocol non-adherence and any statistical methods to handle missing data

Participants will have a 30-s time limit to answer each knowledge item. This limit is to prevent participants from searching for answers on the Internet. If the participant times out, they will receive a missing value of 9. This missing value will be recoded as an incorrect answer to the knowledge item, since the participant could not correctly answer the question in the allotted time.

#### Plans to give access to the full protocol, participant level-data, and statistical code

This document is the full protocol. Anyone interested in other data or documentation should contact the corresponding author.

### Oversight and monitoring

#### Composition of the coordinating center and trial steering committee

The trial will be overseen by a trial steering committee (TSC). The TSC will have an independent chairperson and members but also includes the trial collaborators. Two TSC meetings are planned.

#### Adverse event reporting and harms

It is unlikely there will be adverse events given the online format of the trial. The auditing of trial conduct will be addressed in the two TSC meetings.

#### Dissemination plans

We will disseminate the study findings through journal publications and conference presentations.

## Discussion

There is a critical need for public health actors to disseminate scientific information about the prevention of COVID-19, especially in the absence of a vaccine. To this end, we have produced a short, animated E-E video to improve knowledge of, and behavioral intent, toward preventive COVID-19 behaviors. In this study, we propose to evaluate the effectiveness of our E-E video using three innovative approaches.

First, we will use state-of-the-art online technology to implement our multi-site randomized controlled trial. The ProA platform will enable us to rapidly recruit a large and diverse number of participants who speak different languages and live in different countries in Europe, North America, and Latin America. Second, we will implement the study on the Gorilla platform, which is designed to host and facilitate experimental trials in behavioral research. The Gorilla platform will enable us to randomize at two different levels: (1) participants will be randomized to the E-E intervention, the APC, or the control arm; (2) within each arm, participants will then be randomized to receive a control list or treatment list. Further, the list experiments will also be randomly ordered to avoid order effects. All randomization will be performed automatically by the Gorilla randomization algorithm, which demonstrates the platform’s potential to implement innovative trial designs and concepts.

Third, we will use a list experiment as the primary outcome of this study. Given the unprecedented nature of the COVID-19 outbreak, and the media attention it has received, we assume that participants will already be primed to give socially acceptable responses to questions about COVID-19. In public, individuals will say they will adhere to social distancing recommendations. But in private, they may attend secret “corona parties” [19]. The list experiment gets around the problem of eliciting untruthful answers to socially desirable questions. Further, we have designed the trial in a way, making full use of the Gorilla features, to measure the magnitude of social desirability bias toward the COVID-19 questions.

We expect that our study will make important contributions to the E-E literature. Importantly, lessons learned can help us to improve the design of E-E videos to disseminate public health information for future pandemics. And we may hopefully demonstrate that our E-E video, which has been viewed more than a million times on social media thus far, will have contributed to current public health efforts to keep people safe and reduce the spread of COVID-19. Our results will also guide future E-E strategies to support the long-term COVID-19 response, as countries are easing first-wave lockdown or reimplementing second-wave lockdowns.

### Trial status

The trial is currently recruiting on the ProA website. The first 50 participants were randomized to the pilot phase of the trial on March 23, 2020. A structured summary of the protocol was published on June 3, 2020 [9]. Full recruitment began on June 5, 2020. Thus far, 14,800 surveys have been completed. Recruitment is due to end June 25, 2020. The trial was registered on the German Clinical Trials Register (www.drks.de) on May 12, 2020, with registration number #DRKS00021582.

## Data Availability

Data will be collected and stored on the Gorilla platform. The study investigators own and have complete control of the research data, which can be accessed at any time. For statistical analysis, the data will be downloaded and safely stored on a computing system maintained by the University of Heidelberg.
